# Expiratory flow limitation under moderate hypobaric hypoxia does not influence ventilatory responses during incremental running in endurance runners

**DOI:** 10.14814/phy2.13996

**Published:** 2019-02-03

**Authors:** Yinhang Cao, Yuhei Ichikawa, Yosuke Sasaki, Takeshi Ogawa, Tsutomu Hiroyama, Yasushi Enomoto, Naoto Fujii, Takeshi Nishiyasu

**Affiliations:** ^1^ Faculty of Health and Sport Sciences University of Tsukuba Ibaraki Japan; ^2^ Faculty of Economics Niigata Sangyo University Kashiwazaki Japan; ^3^ Department of Physical Education Osaka Kyoiku University Osaka Japan

**Keywords:** Aerobic capacity, airflow limitation, altitude, endurance performance, respiration

## Abstract

We tested whether expiratory flow limitation (EFL) occurs in endurance athletes in a moderately hypobaric hypoxic environment equivalent to 2500 m above sea level and, if so, whether EFL inhibits peak ventilation ($\dot{V}$E_peak_), thereby exacerbating the hypoxia‐induced reduction in peak oxygen uptake ($\dot{V}$O_2peak_). Seventeen young male endurance runners performed incremental exhaustive running on separate days under hypobaric hypoxic (560 mmHg) and normobaric normoxic (760 mmHg) conditions. Oxygen uptake ($\dot{V}$O_2_), minute ventilation ($\dot{V}$E), arterial O_2_ saturation (SpO_2_), and operating lung volume were measured throughout the incremental exercise. Among the runners tested, 35% exhibited EFL (EFL group, *n* = 6) in the hypobaric hypoxic condition, whereas the rest did not (Non‐EFL group, *n* = 11). There were no differences between the EFL and Non‐EFL groups for $\dot{V}$E_peak_ and $\dot{V}$O_2peak_ under either condition. Percent changes in $\dot{V}$E_peak_ (4 ± 4 vs. 2 ± 4%) and $\dot{V}$O_2peak_ (−18 ± 6 vs. −16 ± 6%) from normobaric normoxia to hypobaric hypoxia also did not differ between the EFL and Non‐EFL groups (all *P* > 0.05). No differences in maximal running velocity, SpO_2_, or operating lung volume were detected between the two groups under either condition. These results suggest that under the moderate hypobaric hypoxia (2500 m above sea level) frequently used for high‐attitude training, ~35% of endurance athletes may exhibit EFL, but their ventilatory and metabolic responses during maximal exercise are similar to those who do not exhibit EFL.

## Introduction

At high altitude, peak oxygen uptake ($\dot{V}$O_2peak_) is lower than at sea level due to a reduction in the amount of oxygen inspired (Stenberg et al. 1966; Lawler et al. 1988; Martin and O'Kroy 1993). However, this reduction in $\dot{V}$O_2peak_ is characterized by large interindividual variations (Young et al. 1985; Fulco et al. 1998; Chapman et al. 1999, 2011; Ogawa et al. 2007; Chapman 2013), with individuals showing greater reductions in $\dot{V}$O_2peak_ exhibiting smaller increases in ventilation in response to hypoxia (Lawler et al. 1988; Gavin et al. 1998; Ogawa et al. 2007; Chapman 2013). The association between ventilation and $\dot{V}$O_2peak_ seems plausible, given that lower ventilation can reduce alveolar O_2_ partial pressure, which would make for a smaller gradient for O_2_ diffusion across the alveolar‐capillary membrane (Harms and Stager 1995; Calbet et al. 2003; Ogawa et al. 2007, 2010), ultimately reducing arterial O_2_ saturation (SpO_2_). As for the determinant driving the individual variation in ventilatory response during hypoxic exercise, we demonstrated that a greater hypoxic ventilatory response, as assessed under resting conditions, correlates with a greater increase in ventilation during maximal exercise under moderate hypobaric hypoxia equivalent to an altitude of 2500 m (Ogawa et al. 2007). However, based on the *R* ^2^ values provided in our previous study (Ogawa et al. 2007), the hypoxic ventilatory response explains only about 30–40% of the individual differences in ventilation. Therefore, one or more other factors appear to play a critical role in determining individual differences in the ventilatory response under these conditions.

During maximal or near maximal exercise, the ventilatory responses of some endurance athletes may be restricted as a consequence of a mechanical limitation classically termed expiratory flow limitation (EFL) (Johnson et al. 1992; Derchak et al. 2002; Dominelli and Sheel 2012). Chapman et al. (1998) reported that endurance athletes with EFL were less able to increase peak ventilation ($\dot{V}$E_peak_) than their counterparts under normobaric hypoxia equivalent to about 1000 m above sea level. However, it should be noted that their study tested responses under normobaric hypoxic conditions, which is different from actual high‐altitude conditions (i.e., hypobaric hypoxia) wherein airflow resistance is reduced due to the lower air density. The reduced air density associated with high‐altitude exposure may alleviate EFL. Consistent with that idea, ~80% reduction in gas density relative to normobaria achieved by inhalation of a helium‐oxygen (He–O_2_) gas mixture greatly increases pulmonary ventilation in individuals who develop EFL (McClaran et al. 1999). As far as we know, EFL under hypobaric hypoxic conditions has been assessed in only one study, which demonstrated that EFL occurs in 50% of endurance athletes under mildly hypobaric hypoxic conditions equivalent to 1545 m above sea level (Foster et al. 2014) wherein air density is reduced by 16% relative to sea level. However, it remains to be determined whether EFL occurs in a moderate hypobaric hypoxic environment equivalent to 2500 m above sea level, which is an altitude frequently employed by athletes for high‐altitude training (Chapman et al. 2014). The occurrence of EFL under the moderate hypobaric hypoxia at 2500 m may be lower than the 50% reported in mild hypobaric hypoxia at 1545 m (Foster et al. 2014), as the reduction in air density is greater at 2500 m than 1545 m (25% vs. 16%).

In this study, therefore, we tested the hypothesis that less than 50% of competitive endurance runners would exhibit EFL when exposed to moderate hypobaric hypoxic conditions equivalent to 2500 m above sea level, and that athletes with EFL would not increase $\dot{V}$E_peak_ under hypobaric hypoxia relative to normobaric normoxia, exacerbating the hypoxia‐induced reduction in $\dot{V}$O_2peak_.

## Materials and Methods

### Ethical approval

This study was carried out in accordance with the Declaration of Helsinki and was approved by the Human Subjects Committee of the University of Tsukuba. All participants provided informed written consent before their participation.

### Participants

Seventeen healthy young male endurance runners participated in this study (means ± SD: age 20 ± 1 years, weight 59 ± 4 kg, height 1.72 ± 0.04 m). We tested males only to avoid any sex‐related differences in pulmonary function (Harms and Rosenkranz 2008). All participants were lowlanders who had not been exposed to hypoxic conditions equal to or above 1000 m for >6 months before participating in this study. All participants were members of the university track‐and‐field team. They were free of cardiopulmonary disease, were not cigarette smokers, and had normal pulmonary function, as indicated by >75% of forced expired volume in 1 s (FEV_1_) relative to forced vital capacity (FVC). Based on the criterion for EFL occurrence described below, there were six participants who exhibited EFL under hypobaric hypoxic conditions (EFL group, *n* = 6) and 11 who did not (Non‐EFL group, *n* = 11). Because the study's focus was on elucidating the effects of EFL on responses during hypobaric hypoxia, all participants were grouped based on the occurrence of EFL under hypobaric hypoxic conditions. Four participants in the EFL group and two in the Non‐EFL group exhibited EFL under normobaric normoxic conditions.

### Preliminary and experimental sessions

Participants completed two preliminary sessions separated by 3–7 days. During the first visit, the participants became familiar with the inspiratory capacity and FVC maneuvers, as well as a progressive running protocol on a treadmill. This procedure was repeated during the second visit. Thereafter, experimental sessions were initiated wherein the participants completed incremental running tests in an environmental chamber (Shimazu; Kyoto, Japan) under normobaric normoxia or hypobaric hypoxia in a counterbalanced manner. The two tests were separated by 3–7 days. The atmospheric pressure was set at a level equivalent to 2500 m above sea level (560 mmHg) in the hypobaric hypoxic condition. This altitude was adopted because it is the one generally chosen for high‐altitude training (Chapman et al. 2014).

### Maximal incremental running tests and pulmonary function assessment

On experimental days, the participants performed a 10‐min warm‐up outside the laboratory. They then entered the environmental chamber where the room temperature was regulated to 20°C, and the room air was continuously ventilated to minimize any increase in CO_2_ inside the chamber. After instrumentation, the participants remained standing on a treadmill for 9 min, during which the time inspiratory capacity maneuver (voluntary maximal inspiratory breathing) was performed two times with a 20‐ to 30‐sec interval in between (Johnson et al. 1992; Weavil et al. 2015). Thereafter, FVC maneuvers were performed with graded effort expirations using the previously described standard protocol (Crapo et al. 1995; Chapman et al. 1998; Guenette et al. 2010; Weavil et al. 2015). The participants then initiated a 3‐min warm‐up at a speed of 160 m min^−1^ on the treadmill, followed by a 3‐min rest. An incremental running test then commenced at a speed of 180 m min^−1^. This initial running speed was increased by 20 m min^−1^ every 2.5 min until volitional fatigue. During the last 30 s of each running stage, the inspiratory capacity maneuver was performed. In addition, FVC maneuvers were performed within 4 min after exhaustion. For safety reasons, a harness was attached to the participants throughout the incremental running. Participants were verbally encouraged throughout the running test. Near the end of the test, expiratory gases were collected into Douglas bags (200–250 L) every minute. We confirmed that all participants met more than two of the following three criteria for $\dot{V}$O_2peak_ (Rice et al. 2000): (1) $\dot{V}$O_2_ reached a plateau and did not increase further despite increases in running speed (<150 mL), (2) peak heart rate (HR) achieved >90% of the age predicted value, and 3) the respiratory exchange ratio was >1.1. Immediately after completion of the incremental running, FVC maneuvers were performed as described above.

### Measurements

Participants breathed through a facemask attached to a two‐way non‐rebreathing valve (Hans Rudolph #2700, Shawnee, KS, USA) with the expiration side connected to a Douglas bag via a bore hose. Inspiratory and expiratory flow as well as expired gases were measured using a mass spectrometer (ARCO‐2000, Arco System, Chiba, Japan). The flow sensor was calibrated using an appurtenant calibration syringe that blew a fixed air volume of 3 L. The O_2_ and CO_2_ sensors were calibrated using standard gases at known concentrations (O_2_ 15.1%, CO_2_ 5.01%, N_2_ balance). The volume of the Douglas bags was determined using a dry gas meter (DC‐5A; Shinagawa; Tokyo, Japan). That information was used to assess $\dot{V}$E_peak_, $\dot{V}$O_2peak_, and $\dot{V}$CO_2peak_. Other respiratory variables obtained at maximal exercise included tidal volume (TVE), breathing frequency (Fb), partial pressure of end tidal O_2_ (PETO_2_), partial pressure of end tidal CO_2_ (PETCO_2_), and ventilatory equivalents for O_2_ ($\dot{V}$E $\dot{V}O_{2}^{-1}$) and CO_2_ ($\dot{V}$E $\dot{V}{\text{CO}}_{2}^{-1}$). SpO_2_ was determined using a forehead pulse oximeter (N‐595; Nellcor, Hayward, CA, USA). HR was measured using a HR monitor (RS400, POLAR, Finland). Rating of perceived exertion (RPE) was measured at each running stage using Borg's scales (Borg 1982). All raw data collected during the incremental running tests were recorded continuously at 200 Hz (PowerLab/16SP model ML 796, ADInstruments, Colorado Springs, CO) and stored on a computer for subsequent data analysis (Lab Chart 6, ADInstruments).

### Data analysis

**Figure 1:**
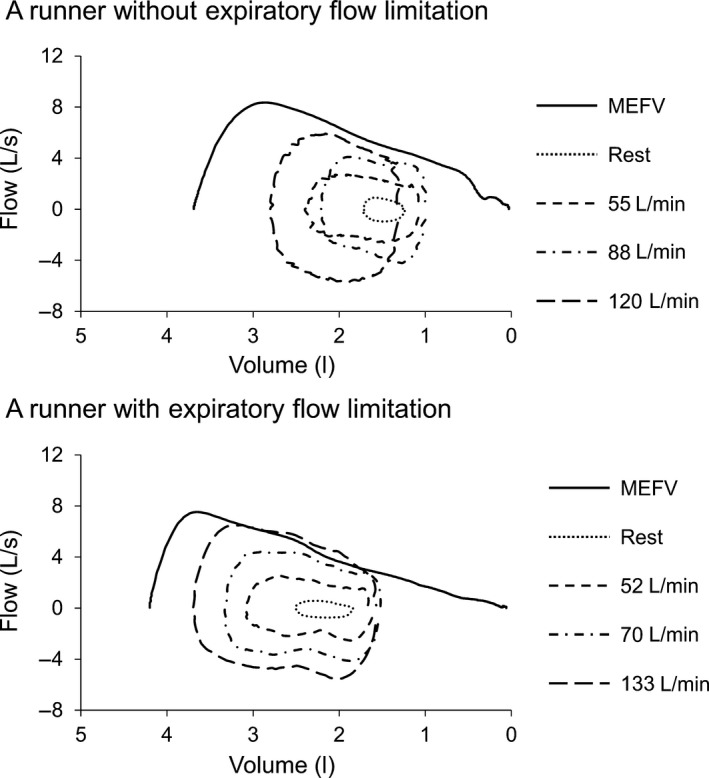
Flow volume loops during an incremental running exercise obtained from a representative runner with or without expiratory flow limitation (EFL) under hypobaric hypoxic conditions. The maximal expiratory flow volume (MEFV) curve is denoted by a thick black line. The circular traces represent tidal flow‐volume loops at rest and during exercise with the indicated minute ventilation levels.

**Figure 2:**
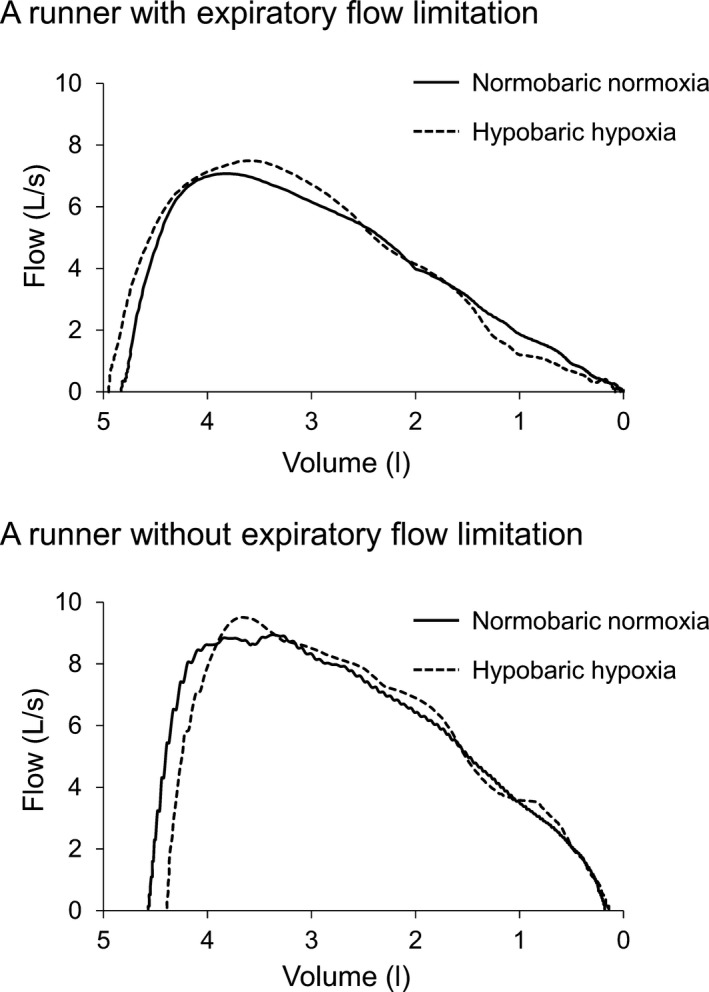
Maximal expiratory flow volume (MEFV) curves under normobaric normoxic and hypobaric hypoxic conditions obtained from a representative runner with or without expiratory flow limitation (EFL).

Pulmonary function indices including FVC, FEV_1_, FEV_1_ FVC^−1^, peak expiratory flow rate (PEFR), and maximal expiratory flow between 25% and 75% of expiration (MEF_25–75_) were obtained from the FVC maneuver as was done previously (Babb 1997; Chapman et al. 1998). Maximal expiratory flow volume (MEFV) curves were constructed based on the highest FVC and FEV_1_ obtained (Duke et al. 2014; Weavil et al. 2015). At each stage (pre‐exercise rest and each running velocity), 10–15 tidal breaths were averaged and inserted into the MEFV curve to provide a representative tidal flow volume loop to determine the degree of EFL (Chapman et al. 1998; Dominelli et al. 2011). The percentage of TVE either reaching or exceeding the boundary of the MEFV curve was determined (Johnson et al. 1992; Dueck 2000), and a value >5% was considered EFL (Derchak et al. 2000). Figure 1 illustrates the rest and exercise flow volume loops plotted relative to the MEFV curve under hypobaric hypoxic conditions in a representative participant from the Non‐EFL or EFL group. Figure 2 illustrates the MEFV curve in a representative participant from the Non‐EFL or EFL group under both normobaric normoxic and hypobaric hypoxic conditions. Operating lung volumes, including both expiratory reserve volume (ERV) and inspiratory reserve volume (IRV) were determined (Taylor et al. 2013; Weavil et al. 2015). ERV was calculated by subtracting the inspiratory capacity from FVC (Johnson et al. 1999), whereas IRV was calculated as ERV plus TVE (Guenette and Sheel 2007). ERV and IRV were expressed as %FVC (Guenette and Sheel 2007). Ventilatory capacity ($\dot{V}$Ecap) was calculated as maximal breathing frequency (estimated from the minimal expiratory and inspiratory durations) multiplied by the TVE, as described previously (Dominelli et al. 2012; Molgat‐Seon et al. 2017). Percent ventilatory capacity utilization during maximal exercise ($\dot{V}$E_peak_ $\dot{V}$Ecap^−1^) was also assessed (Tanner et al. 2014). SpO_2_ was not successfully recorded from four Non‐EFL participants and one EFL participant due to technical issues under both normobaric normoxic and hypobaric hypoxic conditions.

### Statistical analysis

Data are presented as means ± SD. Two‐way mixed‐model ANOVA was used for pulmonary function indices (FVC, FEV_1_, FEV_1_ FVC^−1^, PEFR, MEF_25–75_, $\dot{V}$Ecap, and $\dot{V}$E_peak_ $\dot{V}$Ecap^−1^) (Table 2) and maximal exercise values ($\dot{V}$O_2peak_, $\dot{V}$CO_2peak_, $\dot{V}$E_peak_, HR_peak_, TVE, Fb, PETO_2_, PETCO_2_, $\dot{V}$E $\dot{V}O_{2}^{-1}$, $\dot{V}$E $\dot{V}{\text{CO}}_{2}^{-1}$, SpO_2_, RPE, and maximal running velocity) (Table 3) with factors of group (EFL and Non‐EFL) and condition (normobaric normoxia and hypobaric hypoxia). ERV and IRV during the incremental running tests were analyzed using a three‐way mixed‐model ANOVA with factors of group (EFL and Non‐EFL), condition (normobaric normoxia and hypobaric hypoxia), and exercise intensity (rest, 40%, 60%, 80%, and 100% $\dot{V}$O_2peak_). After determining the main effects, post hoc multiple comparisons were made using the Holm–Bonferroni method. In addition, descriptive characteristics were compared between the Non‐EFL and EFL groups using two‐tailed unpaired *t*‐tests. Values of *P *< 0.05 were considered statistically significant. Cohen's *d* effect sizes were calculated to identify the magnitude of differences between the two groups (Cohn 1988). Pearson product moment correlations were determined for the association between the EFL magnitude and percent changes in $\dot{V}$E_peak_, SpO_2_, and $\dot{V}$O_2peak_ in the EFL group. The SPSS 25 statistical software package for Windows (IBM, Armonk, NY, USA) was used for all statistical analyses.

## Results

**Figure 3:**
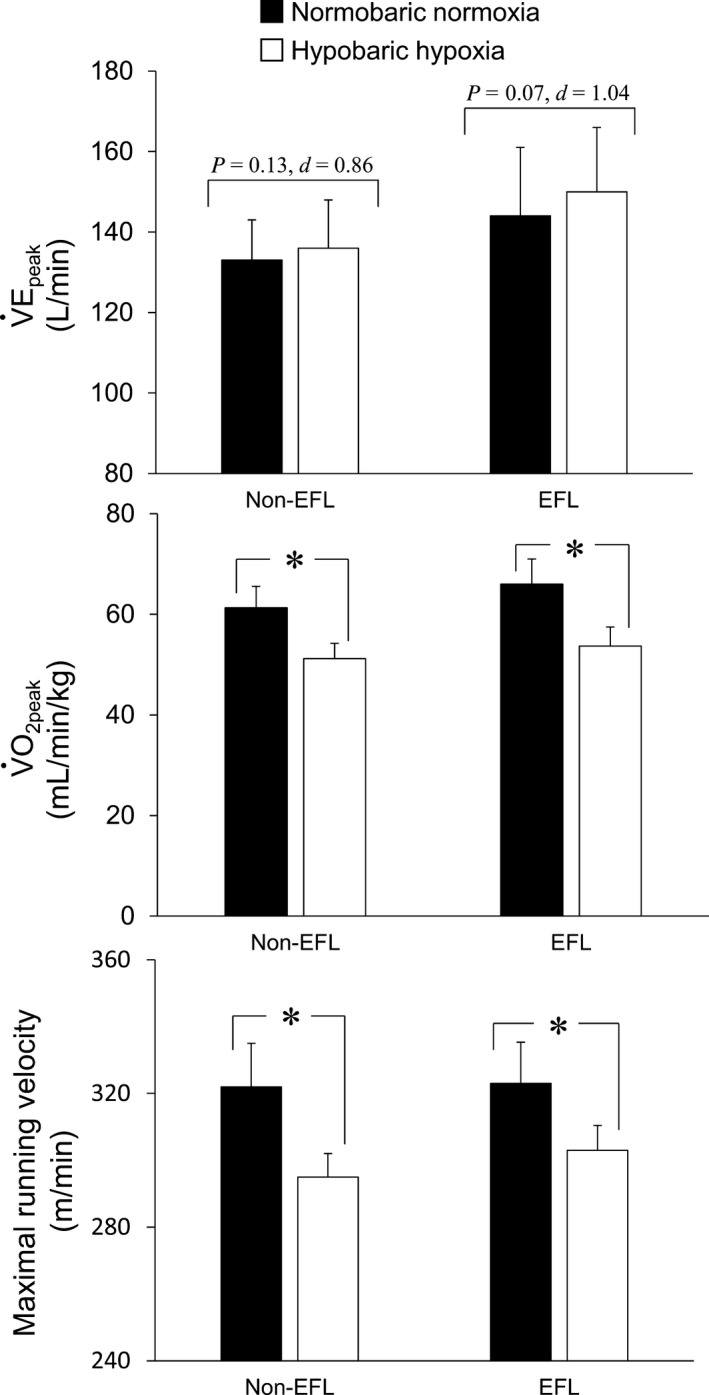
Peak ventilation ($\dot{V}$E_peak_), peak oxygen uptake ($\dot{V}$O_2peak_), and maximal running velocity under normobaric normoxic and hypobaric hypoxic conditions. Non‐EFL, runners without expiratory flow limitation; EFL, runners with expiratory flow limitation. *P < 0.05 hypobaric hypoxia versus normobaric normoxia.

**Figure 4:**
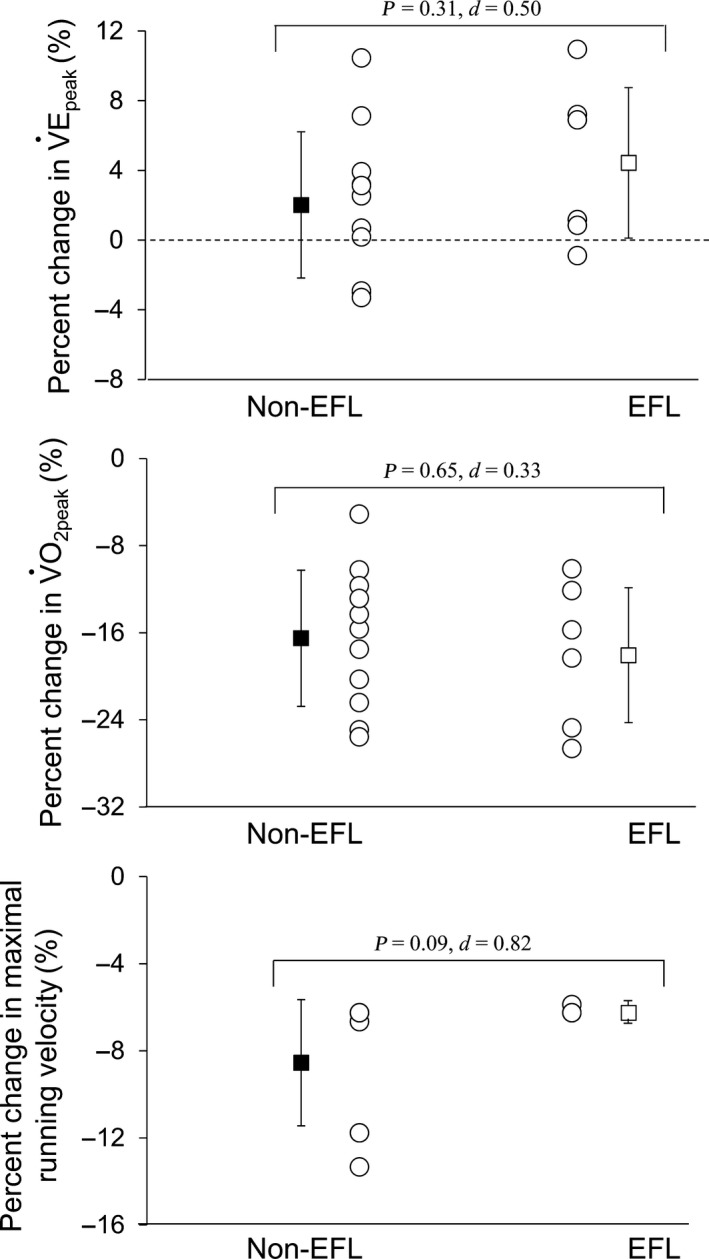
Percent changes in peak ventilation ($\dot{V}$E_peak_), peak oxygen uptake ($\dot{V}$O_2peak_), and maximal running velocity from normobaric normoxic to hypobaric hypoxic conditions in the two groups. Both individual (white circles) and mean (black and white squares) values are presented. Non‐EFL, runners without expiratory flow limitation (black square); EFL, runners with expiratory flow limitation (white square).

**Figure 5:**
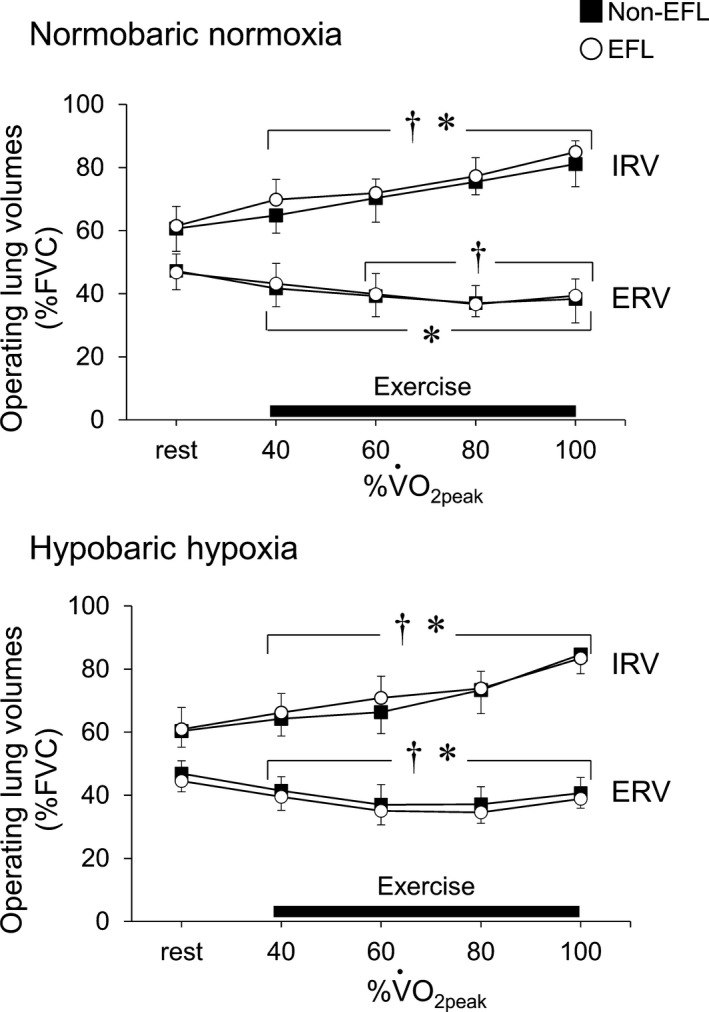
Changes in operating lung volume assessed from rest to maximal exercise under normobaric normoxic and hypobaric hypoxic conditions. ERV, expiratory reserve volume; IRV, inspiratory reserve volume; FVC, forced vital capacity; Non‐EFL, runners without expiratory flow limitation; EFL, runners with expiratory flow limitation; %VO_2peak_, percentage of peak oxygen uptake obtained under normobaric normoxic and hypobaric hypoxic conditions. *P < 0.05. exercise versus rest in the Non‐EFL group; ^†^ *P* < 0.05 exercise versus rest in the EFL group.

Based on the MEFV data, 6 (35%) endurance runners showed EFL under hypobaric hypoxia (15–62%), whereas the remaining 11 (65%) did not (Table 1). Comparison of the two groups revealed that runners with and without EFL had similar aerobic capacities and running performances under normobaric normoxic conditions, as judged from their $\dot{V}$O_2peak_ (66 ± 3 vs. 61 ± 4 mL/min/kg) and maximal running velocity (323 ± 7 vs. 322 ± 13 m min^−1^) (all *P* > 0.05, Fig. 3). Under normobaric normoxic conditions, runners in the Non‐EFL and EFL groups also had similar $\dot{V}$E_peak_ (133 ± 10 vs. 144 ± 17 L min^−1^, *P* = 0.13, *d* = 0.86, Fig. 3). Despite the presence of EFL under hypobaric hypoxic conditions, endurance runners in the EFL, and Non‐EFL groups showed similar $\dot{V}$O_2peak_ (54 ± 4 vs. 51 ± 5 mL/min/kg, *P* > 0.05) and maximal running velocity (303 ± 7 vs. 295 ± 12 m min^−1^_,_ *P* > 0.05). $\dot{V}$E_peak_ (150 ± 16 vs. 136 ± 12 L min^−1^, *P* = 0.07, *d* = 1.04) tended to be higher in the EFL than Non‐EFL group (Fig. 3). The percent change from normobaric normoxia to hypobaric hypoxia was similar in the Non‐EFL and EFL groups for $\dot{V}$E_peak_ (2 ± 4 vs. 4 ± 4%, *P* = 0.31, *d* = 0.50) and $\dot{V}$O_2peak_ (−16 ± 6 vs. −18 ± 6%, *P* = 0.65, *d* = 0.33) (Fig. 4). In contrast, the percent change in maximal running velocity tended to be larger in the Non‐EFL than EFL group (−8 ± 3 vs. −6 ± 0%, *P* = 0.09, *d* = 0.82) (Fig. 4). No correlative relationships were observed for EFL magnitude versus the percent changes in $\dot{V}$E_peak_ (*r* = 0.10; *P* > 0.05), SpO_2_ (*r* = 0.05; *P* > 0.05) or $\dot{V}$O_2peak_ (*r* = 0.61; *P* > 0.05) from normobaric normoxia to hypobaric hypoxia in the EFL group. Other variables did not differ between the two groups in either normobaric normoxia or hypobaric hypoxia (all *P* > 0.05), with the exception that PETO_2_ was higher (*P* < 0.05) and PETCO_2_ tended to be lower (*P* = 0.12) in the EFL than Non‐EFL group in hypobaric hypoxia (Table 3). In addition, based on ERV and IRV, there were no between‐group differences in operating lung volumes under either condition (*P* > 0.05) (Fig. 5).

**Table 1:** Participant characteristics

	Non‐EFL	EFL
Age (years)	21 ± 1	20 ± 1
Weight (kg)	59.2 ± 4.2	59.1 ± 3.0
Height (m)	1.71 ± 0.05	1.73 ± 0.03

Values are means ± SD; Non‐EFL, runners without expiratory flow limitation (*n* = 11); EFL, runners with expiratory flow limitation (*n* = 6).

All descriptive characteristics (Table 1) and pulmonary function indices (Table 2) were similar between the Non‐EFL and EFL groups, though participants with EFL showed lower FEV_1_ FVC^−1^ and $\dot{V}$Ecap, and higher $\dot{V}$E_peak_ $\dot{V}$Ecap^−1^ under both normobaric normoxic and hypobaric hypoxic conditions (*P* < 0.05) (Table 2). There were no differences in these pulmonary function indices between normobaric normoxia and hypobaric hypoxia for either the EFL or Non‐EFL group (all *P* > 0.05) (Table 2).

**Table 2:** Pulmonary function indices assessed under normobaric normoxia and hypobaric hypoxia

	Group	Normobaric Normoxia	Hypobaric Hypoxia	%change	%predicted
FVC (L)	Non‐EFL	4.45 ± 0.39	4.39 ± 0.40	−1.2 ± 4.4	101 ± 9
	EFL	4.44 ± 0.60	4.34 ± 0.57	−2.0 ± 4.2	99 ± 12
FEV_1_ (L)	Non‐EFL	4.00 ± 0.33	3.90 ± 0.40	−2.6 ± 5.9	91 ± 8
	EFL	3.71 ± 0.37	3.53 ± 0.41	−4.9 ± 3.2	81 ± 8
FEV_1_ FVC^−1^ (%)	Non‐EFL	90 ± 3	89 ± 3	−1.2 ± 4.4	90 ± 3
	EFL	84 ± 5*	82 ± 7*	−2.4 ± 7.3	83 ± 7*
PEFR (L sec^−1^)	Non‐EFL	9.3 ± 1.5	9.3 ± 1.5	2.0 ± 11.1	–
	EFL	8.5 ± 1.0	8.5 ± 0.7	1.4 ± 7.3	–
MEF_25–75_ (L sec^−1^)	Non‐EFL	8.6 ± 1.5	8.8 ± 1.1	−1.0 ± 12.0	–
	EFL	7.8 ± 1.1	7.7 ± 1.0	0.5 ± 8.8	–
$\dot{V}$Ecap (L min^−1^)	Non‐EFL	232 ± 24	235 ± 22	1.6 ± 4.4	–
	EFL	195 ± 28*	193 ± 23*	−0.9 ± 3.4	–
$\dot{V}$E_peak_ $\dot{V}$Ecap^−1^ (%)	Non‐EFL	58 ± 8	59 ± 10	0.4 ± 5.3	–
	EFL	75 ± 8*	79 ± 8*	5.3 ± 3.5	–

Values are means ± SD; Non‐EFL, runners without expiratory flow limitation (*n* = 11); EFL, runners with expiratory flow limitation (*n* = 6). FVC, forced vital capacity; FEV_1_, forced expired volume in 1 sec; PEFR, peak expiratory flow rate; MEF_25–75_, maximal expiratory flow between 25% and 75% of forced vital capacity; $\dot{V}$Ecap, theoretical ventilatory capacity; $\dot{V}$E_peak_ $\dot{V}$Ecap^−1^, percent of ventilatory capacity utilization; *P < 0.05 EFL versus Non‐EFL.

## Discussion

The major findings of this study are that: 1) EFL occurred in 35% (6 of 17 runners) of competitive endurance runners tested under moderate hypobaric hypoxic conditions equivalent to 2500 m above sea level; 2) the percent changes in $\dot{V}$E_peak_ and $\dot{V}$O_2peak_ from normobaric normoxia to hypobaric hypoxia did not differ between the EFL and Non‐EFL groups. These findings suggest that ~35% of competitive endurance runners would exhibit EFL under moderate hypobaric hypoxia, but this EFL does not appear to affect their ventilatory responses to moderate hypobaric hypoxic stimulation.

### Effects of EFL under moderate hypobaric hypoxia

This study was designed to elucidate the incidence of EFL in trained endurance athletes under moderate hypobaric hypoxia, a condition that is typically used for high‐altitude training, and whether EFL affects physiological responses under these conditions. Among the runners tested, 35% exhibited EFL under the moderate hypobaric hypoxia. As we originally hypothesized, this percentage is smaller than that reported in an earlier study wherein ~50% of elite Kenyan runners exhibited EFL under mild hypobaric hypoxia equivalent to 1545 m above sea level (Foster et al. 2014). At first glance, the lower incidence of EFL in moderate hypobaric hypoxia in this study could be explained by the greater reduction in air density, which would somewhat reduce mechanical limitation and thus EFL. However, our results show that the incidence of EFL did not differ between normobaric normoxia and hypobaric hypoxia (35% vs. 35%). A future study with a larger sample size will be required to clearly elucidate the relationship between the incidence of EFL and the air density reduction associated with hypobaric hypoxia.

We do not know why in this study some endurance runners showed EFL, whereas others did not. Given that age, weight, height, $\dot{V}$O_2peak_, FVC, and FEV_1_ were similar between the groups under both normobaric normoxia and hypobaric hypoxia, these do not appear to explain the occurrence of EFL. However, we did observe that pulmonary expiratory function, as assessed by FEV_1_ FVC^−1^, was lower in EFL than Non‐EFL athletes under both normobaric normoxia and hypobaric hypoxia. In addition, $\dot{V}$Ecap, which reflects an individual's intrinsic respiratory anatomy (e.g., lung and airway size) (Dominelli et al. 2015), was lower in the EFL than Non‐EFL group (Table 2). Thus, a difference in airway structural characteristics between EFL and Non‐EFL athletes may contribute to the occurrence of EFL.

Alternatively, $\dot{V}$E_peak_ tended to be higher in the EFL than Non‐EFL group in hypobaric hypoxia (Fig. 3), and gas exchange also appears to be greater in the EFL group, as evidenced by our finding that the EFL group tended to exhibit higher PETO_2_ and lower PETCO_2_ in hypobaric hypoxia (Table 3). It may be, therefore, that the EFL group can increase ventilation to a greater extent than the Non‐EFL group, and reach a level where mechanical limitation occurs. A similar idea was also proposed in several earlier studies (Johnson et al. 1992; Chapman et al. 1998; Weavil et al. 2015). This possibility will need to be directly evaluated in the future.

**Table 3:** Variable obtained at maximal exercise

	Group	Normobaric normoxia	Hypobaric hypoxia	%change
$\dot{V}$CO_2peak_ (L min^−1^)	Non‐EFL	3.93 ± 0.39	3.50 ± 0.47*	−11.0 ± 9.0
	EFL	4.16 ± 0.28	3.64 ± 0.42*	−12.6 ± 6.4
PETO_2_ (mmHg)	Non‐EFL	116 ± 5	78 ± 2*	−32.6 ± 7.9
	EFL	118 ± 3	82 ± 4* ^,^ †	−30.4 ± 3.2
PETCO_2_ (mmHg)	Non‐EFL	38 ± 5	33 ± 3*	−11.1 ± 16.9
	EFL	38 ± 2	31 ± 2*	−18.5 ± 6.7
TVE (L)	Non‐EFL	2.08 ± 0.28	2.01 ± 0.29	−3.2 ± 7.9
	EFL	2.07 ± 0.29	1.99 ± 0.31	−3.8 ± 3.4
Fb (breaths min^−1^)	Non‐EFL	65 ± 7	68 ± 8*	4.7 ± 4.4
	EFL	69 ± 9	74 ± 9	6.6 ± 6.4
$\dot{V}$E $\dot{V}O_{2}^{-1}$	Non‐EFL	37 ± 4	45 ± 5*	22.2 ± ± 8.5
	EFL	38 ± 4	48 ± 2*	28.3 ± 10.8
$\dot{V}$E $\dot{V}{\text{CO}}_{2}^{-1}$	Non‐EFL	34 ± 3	39 ± 4*	15.5 ± 10.0
	EFL	35 ± 3	41 ± 2*	19.9 ± 9.0
SpO_2_ (%)	Non‐EFL	91 ± 4 (*n* = 7)	76 ± 3* (*n* = 7)	−15.7 ± 3.4
	EFL	91 ± 2 (*n* = 5)	77 ± 2* (*n* = 5)	−16.0 ± 1.7
HR_peak_ (beats min^−1^)	Non‐EFL	192 ± 7	187 ± 7*	−2.8 ± 1.5
	EFL	191 ± 11	185 ± 9*	−2.9 ± 1.1
RPE	Non‐EFL	19 ± 1	19 ± 1	3.1 ± 3.4
	EFL	18 ± 1	19 ± 1	5.4 ± 2.2

Values are means ± SD; Non‐EFL, runners without expiratory flow limitation (*n* = 11); EFL, runners with expiratory flow limitation (*n* = 6). $\dot{V}$CO_2peak_, peak carbon dioxide production; PETO_2_, partial pressure of end tidal O_2_; PETCO_2_, partial pressure of end tidal CO_2_; TVE, tidal volume; Fb, breathing frequency; $\dot{V}E$ $\dot{V}O_{2}^{-1}$, ventilatory equivalent for O_2_; $\dot{V}E$ $\dot{V}{\text{CO}}_{2}^{-1}$, ventilatory equivalent for CO_2_; SpO_2_, arterial O_2_ saturation; HR_peak_, peak heart rate; RPE, rating of perceived exertion; *P < 0.05 normobaric normoxia versus hypobaric hypoxia; ^†^ *P* < 0.05 EFL versus Non‐EFL.

To assess the influence of EFL on ventilatory responses during exercise in moderate hypobaric hypoxia, we compared the ventilatory and metabolic responses between the EFL and Non‐EFL groups. In contrast to our hypothesis, the percent changes in $\dot{V}$E_peak_ from normobaric normoxia to hypobaric hypoxia were similar between the EFL and Non‐EFL groups (Fig. 4). Moreover, no between‐group difference was observed for the percent decrement in $\dot{V}$O_2peak_ (Fig. 4). These results suggest that ventilatory and metabolic responses during maximal exercise are not constrained by EFL in a moderately hypobaric hypoxic environment (e.g., 2500 m above sea level).

An earlier study showed that breathing He–O_2_ can increase PEFR, MEF_25–75_, and the size of the MEFV curve, thereby decreasing EFL. As a consequence, breathing He–O_2_ increases $\dot{V}$E_peak_ in athletes who exhibit EFL (Guenette and Sheel 2007). This raises the possibility that the ~25% lower air density observed under the hypobaric hypoxic conditions could increase the PEFR, MEF_25–75_, and the MEFV curve, but this was not the case in this study (Fig. 2). Along these lines, neither the incidence of EFL nor any of the tested pulmonary function indices (Table 2) differed between normobaric normoxia and hypobaric hypoxia in this study. Because the reduction in air density in the moderately hypobaric hypoxic environment used in this study was not as low as in He–O_2_ (~25% vs. 80% of normal air), it may be that a greater reduction in air density is required to observe a clear effect on EFL and pulmonary function, but this remains to be tested.

We previously demonstrated that around 30–40% of the individual differences in ventilation attained during maximal exercise under moderate hypobaric hypoxic conditions (2500 m) is attributable to differences in ventilatory sensitivity to hypoxia (Ogawa et al. 2007). This means that around 60–70% of individual variation in ventilation is explained by one or more other factors. Our results indicate that EFL does not explain the individual differences in ventilation. Alternatively, high‐intensity exercise would evoke metaboreceptor activation in the active muscles, and that response may be enhanced by breathing hypoxic air due to a greater anaerobic metabolism in the active muscles. High‐intensity exercise also increases body temperature due to heat production by the active muscles (Kenny et al. 2003). Given that both metaboreceptor activation and hyperthermia can increase ventilation (Kaufman and Forster 1996; White 2006; Fujii et al. 2008; Sheel and Romer 2012; Tsuji et al. 2016), individual differences in the magnitudes of these responses may contribute to the interindividual differences in ventilation seen during maximal exercise under moderate hypobaric hypoxia. Both of these possibilities remain to be tested.

### Operating lung volumes

Previous studies showed that breathing He–O_2_ reduces ERV compared to a gas mixture without helium (Babb 1997; McClaran et al. 1999; Quon et al. 2015). This suggests that low airway resistance can modulate ERV. As far as we know, we are the first to assess whether reduction in airway resistance associated with exposure to moderate hypobaric hypoxia alters operating lung volume relative to normobaric normoxia. We found that operating lung volumes did not differ between the two conditions in either group (Fig. 5). A reduction in airway resistance associated with exposure to moderate hypobaric hypoxia thus does not appear to be sufficient to alter ERV.

During low‐to‐moderate intensity exercise in this study, ERV decreased from resting levels under normobaric normoxia in both groups (Fig. 5). This is consistent with earlier observations (Pellegrino et al. 1993; Mota et al. 1999; Smith et al. 2017). The reduction in ERV is thought to aid inspiration by optimizing diaphragmatic length, thereby permitting elastic recoil of the chest wall (Henke et al. 1988). During high‐ to maximal‐intensity exercise in this study, ERV did not differ between the EFL and Non‐EFL groups and remained lower than resting levels under normobaric normoxia (Fig. 5). This is in contrast to the study from Dominelli et al. (2011) who reported that the ERV was higher in Non‐EFL than EFL females during high‐ to maximal‐intensity exercise under normobaric normoxic conditions. Those investigators speculated that the increase in ERV is due to individuals approaching the mechanical limits of their ability to generate expiratory flow, and ERV must therefore be increased to avoid EFL. The discrepancy between the present and previous studies may be due to differences in gender (males vs. females) and/or the training status of the participants (trained runners vs. untrained individuals), as well as the tested conditions (hypobaric hypoxia vs. normobaric normoxia).'

## Conclusions

Our results suggest that in a moderate hypobaric hypoxic environment equivalent to 2500 m above sea level, which is regularly used for high‐altitude training, ~35% of trained endurance runners may exhibit EFL. However, this EFL does not appear to affect ventilatory responses or respiratory mechanics during incremental running in a moderately hypobaric hypoxic environment wherein air density is reduced by 25% relative to sea level.

## Conflict of Interest

No conflicts of interest, financial or otherwise, are declared by the author(s).
